# Subjective Social Status, Area Deprivation, and Gender Differences in Health among Chinese Older People

**DOI:** 10.3390/ijerph19169857

**Published:** 2022-08-10

**Authors:** Xi Chen, Jean Woo, Ruby Yu, Gary Ka-Ki Chung, Wei Yao, Eng-Kiong Yeoh

**Affiliations:** 1 Department of Sociology and Social Policy, Lingnan University, Hong Kong SAR, China; 2 JC School of Public Health and Primary Care, The Chinese University of Hong Kong, Hong Kong SAR, China; 3 CUHK Jockey Club Institute of Ageing, The Chinese University of Hong Kong, Hong Kong SAR, China; 4 CUHK Institute of Health Equity, The Chinese University of Hong Kong, Hong Kong SAR, China; 5 Department of Sociology, The Chinese University of Hong Kong, Hong Kong SAR, China

**Keywords:** subjective social status, area deprivation, physical health, mental health, gender differences, status in the community, older people, Hong Kong

## Abstract

This study examined the gender differences in the main and interactive effects of subjective social status and area deprivation on health among older adults in Hong Kong. Data for this study came from the baseline of MrOs and MsOs studies, including 4000 Chinese men and women ≥ 65 in Hong Kong. Subjective social status was assessed using the MacArthur Scale of subjective social status scale. Our results reaffirm that subjective social status is an independent indicator of health after adjusting for objective SES measures (e.g., education and income). Perceived rank on the community ladder was more closely related to health among older people than was the society ladder, particularly for women. Although area-level social deprivation was not significantly associated with the health of older people, it may moderate the effect of subjective social status on health. Women with a lower perceived status in the community were more likely to experience depressive symptoms but better grip strength when living in more deprived neighborhoods. The findings suggested that subjective social status provides important information for the physical and mental health of the older population. Policymakers may implement interventions to enhance the subjective social status of older adults. Given the greater contribution of relative status in the community to the health of women, these policies and interventions should target to improve women’s perceived status in the community.

## 1. Introduction

The association between social status and health outcomes has been documented in extensive literature in Western [1,2] and Chinese societies [3,4]. People with higher socioeconomic status (SES) typically enjoy better physical and mental health than those with a lower SES. In addition to traditional measures of social status (e.g., education, occupation, and income), subjective social status is a robust predictor of health. People who perceive themselves as having higher status are generally healthier than those who perceive themselves as having lower status [5,6]. Additionally, subjective social status has been frequently found to be more strongly related to health indicators than traditional socioeconomic measures [7,8], perhaps because a person’s assessment of their relative standing in society is a more comprehensive measure of SES than conventional measures. Subjective social status may capture the intangible factors of SES [9], account for changing SES over the lifetime [10], and capture relevant psychological processes, such as interpersonal relative deprivation [11]. However, limited studies have examined whether there are sex differences in the associations between subjective social status and health outcomes. Additionally, we know less about how individual perception of rank on the social hierarchy may interact with area-level SES in predicting health outcomes.

Subjective social status is particularly a strong predictor of health in older adults, as shown in a recent meta-analysis of society and community ladders on health [6]. It may be because that older people, unlike their younger counterparts, are more aware of their social status, and their perceived social status can reflect the cumulative impact of SES throughout their lifetime [3]. Thus, the perceived social status may influence older adults’ lives and future prospects and needs to be considered when measuring SES in older populations. The MacArthur Scale of Subjective Social Status is a widely used measure of subjective social status [12]. Prior studies have found that both the society and community ladders are significantly associated with physical health, mental health, and self-rated health among older people in Hong Kong [9,13]. 

The effect of subjective social status on health may vary between men and women. For example, Adler et al. found that the inverse association between subjective social status and depressive symptoms was stronger among African American women than African American men [14]. Similarly, a recent study in South Africa also revealed that the contribution of subjective social status to inequalities in depressive symptoms was higher for women than men [15]. However, the two studies only considered the society ladder. One study of 300 married couples in the US found that both society and community ladders predicted depressive symptoms for men, but only the community ladder was predictive for depressive symptoms in women. To date, the sex-specific analysis of subjective status and its relationship with health is limited to non-Chinese societies [12,16,17,18]. Little data are available on sex differences in the association between subjective social status and health outcomes in Chinese populations.

Additionally, emerging literature has suggested that regional deprivation is an important determinant for health inequality [19] and an independent indicator of individuals’ health beyond the effect of individual-level SES [20]. Disadvantaged regions may expose their residents to more life stressors and lack material and social resources for them to cope with these stressors [21]. However, findings on the relationship between area deprivation and health outcomes among older people are inconsistent. While some studies found that the association between area deprivation and quality of life may be stronger in old age when people spend more time at home and are more dependent on community-based resources such as community support and health care [22,23,24], other studies found no significant relationship between area-level deprivation and mental well-being among older people [25]. Moreover, area deprivation may condition the association between individual-level SES on health, but the evidence so far is mixed. Some scholars argued that living in deprived areas may reinforce the association between individual socioeconomic position and health [26], consistent with the double jeopardy hypothesis [27]. In other words, people with low social status may be worse off if they reside in disadvantaged areas than living in better-off areas. In contrast, the relative deprivation hypothesis posits that the health of lower SES individuals may be worse if they live in higher SES areas than if they reside in lower SES areas because low SES individuals living in high SES areas may experience more psychosocial stress resulting from upward social comparisons, greater isolation, and difficulty in social integration [28,29].

To our knowledge, no studies have examined how subjective social status and its interaction with area deprivation may affect the health of men and women differently in Chinese societies. The goals of this study were (1) to assess the effect of subjective social status and area-level deprivation on physical and mental health among older adults, (2) to investigate the interactive effect between subjective social status and area deprivation on health, and (3) to examine the gender differences in the main and interactive effects of subjective social status and area deprivation on health status. The health outcomes in the models included mental health (i.e., depressive symptoms), physical health (i.e., grip strength), and health-related quality of life that was measured with the Short Form-2 (SF-12).

There is extensive literature documenting gender differences in health outcomes among older adults in terms of the prevalence of chronic diseases, geriatric syndromes, the aging process itself, and adoption of healthy lifestyles [30,31,32,33].

Based on theoretical propositions and findings of existing studies, we developed the following hypotheses.

**Hypothesis** **1.** *Subjective social status would positively affect physical and mental health among older people.*

**Hypothesis** **2.** *Area-level social deprivation would negatively affect physical and mental health among older adults.*

As for the potential sex difference in the effect of subjective social status, we expected a more significant impact of subjective status on women’s health than men’s. With women typically being more sensitive to affiliation concerns [34,35], women with a lower perceived social status may have limited social networks and support and may be more vulnerable to mental and physical health issues. Thus, we hypothesized the following:

**Hypothesis** **3.** *The relationship between subjective social status and health outcomes may be stronger among women.*

As for the interactive effect between subjective social status and area-level deprivation, we developed the following hypotheses based on the double-jeopardy or relative deprivation hypotheses.

**Hypothesis** **4a.** *Participants with lower subjective social status may experience worse health outcomes if they live in areas with a higher level of social deprivation (double-jeopardy hypothesis).*

**Hypothesis** **4b.** *Participants with lower subjective social status may experience worse health outcomes if they live in areas with a lower level of social deprivation (relative deprivation hypothesis).*

The findings of this research will contribute to a more nuanced understanding of health disparities among older people by revealing how social differentiation at the individual and area levels is translated into health disparities.

## 2. Materials and Methods

### 2.1. Participants and Procedures

Participants for this study came from the MrOs (Hong Kong) and MsOs (Hong Kong) studies, the first large-scale prospective studies on bone health that have followed a cohort of 4000 Chinese men and women aged 65 years and above in Hong Kong since 2001. The method of recruitment consisted of notices placed in housing estates and community centers all over Hong Kong. Stratified sampling was adopted in the study in order to achieve approximately 33% of subjects in each of the three age groups: 65–69, 70–74, and ≥75 years. Those who were unable to walk independently, had a bilateral hip replacement, or were not competent to give informed consent were excluded. Further details about MrOs are published elsewhere [36]. Participants were interviewed at the study site at the Prince of Wales Hospital at baseline, year 2, year 4, and year 14 by trained interviewers. The study was approved by the Clinical Research Ethics Committee of the Chinese University of Hong Kong, which required informed consent to be obtained. All participants signed the study consent form.

### 2.2. Measurements

Information regarding age, sex, marital status, educational level, maximum lifetime income, subjective social status, depressive symptoms, health-related quality of life, and grip strength was obtained as part of a questionnaire administered by trained interviewers. An area deprivation index was also calculated for 18 districts in HK.

#### 2.2.1. Subjective Social Status

Subjective social status was assessed using the MacArthur Scale of subjective social status scale [12]. Participants were asked to place a mark on a picture of an upright ladder with 10 rungs, with the lowest rung indicating the most undesirable and the highest rung indicating the most desirable state with respect to their standing in their self-defined community (known as the “community ladder” [37]). Participants were also asked to rate themselves by placing a mark on a picture of another ladder, the top rung representing people who have the most money, the most education, and the most respected jobs, and the bottom rung representing people at the other extreme (known as the “society ladder” [13]).

#### 2.2.2. Area-Level Social Deprivation Index

The area-level Social Deprivation Index (SDI) is a multi-dimensional measure to quantify the aggregate level of social disadvantages of people residing in a given district. Comparison of health outcomes by the SDI is useful in health inequality monitoring, healthcare planning and resource allocation, and design for community interventions. To measure the area-level social deprivation index (SDI) [38], socioeconomic characteristics of 18 District Council districts in Hong Kong were obtained from the 2016 Hong Kong population by-census [39]. The six socioeconomic domains of SDI included (i) no education (proportion of people with no schooling); (ii) low income (proportion of households with income lower than 50% of median monthly household income by household size); (iii) low occupation (proportion of working people other than “Managers and administrators” among the working population); (iv) divorced population (proportion of people who are divorced or separate); (v) non-nuclear family composition (proportion of households that are not nuclear family); and (vi) family size of two persons (proportion of two-person households). The six indicators were chosen because previous studies have confirmed that no schooling (education), low income (income), non-managerial position (occupation), divorced/separated (marital status), nuclear family (family composition), two-person household (family size) were significantly associated with various health outcomes among Hong Kong residents [38]. A simple summation of the proportions of these six socioeconomic domains was used to estimate the SDI score for each District Council district. We then dichotomized SDI into low (below the mean) and high (equal to or greater than the mean) levels of deprivation. Further details on the construction of SDI are reported elsewhere [38].

#### 2.2.3. Outcomes

Depressive symptoms were assessed with the 15-item Geriatric Depression Scale (GDS-15) [40]. A score of 5 was suggested as the cut-off point to screen for depressive disorders [41]. Health-related quality of life was measured with the Short Form-2 (SF-12), which includes physical and mental domains [42]. Higher scores on the SF-12 indicate a better health-related quality of life in both domains. Grip strength was measured using a dynamometer (JAMAR Hand Dynamometer 5030JO; Sammons Preston, Bolingbrook, IL, USA). Two readings were taken from each side, and the maximum value of the right/left was used for analysis.

### 2.3. Analytical Strategy

All the analyses were performed using Stata 16.0 (StataCorp LLC, College Station, TX, USA). Descriptive statistics were used to characterize the study population, and t/chi-square tests were performed to assess the potential sex differences in variables. A series of regression models were used to examine the association between perceived rank in the society and community ladder and area-level deprivation in mental and physical health. We fit logistic regression for the binary outcome (i.e., depression) and ordinary least squares (OLS) regression models for continuous outcomes (i.e., grip strength, physical and mental domains of quality of life). We first assessed the independent effect of two ladders and area deprivation on mental and physical health after controlling for demographic backgrounds and objective SES measures. We then computed two-way interaction terms between sex with society ladder, community ladder, and area deprivation to examine whether the effect of subjective social status and area-level deprivation on health differed between men and women. Next, we computed the two-way interactions between two ladders and area deprivation to investigate the potential interactive effect between subjective social status and area deprivation on health outcomes. Such analyses were repeated for the subsamples of men and women. Variance inflation factors (VIFs) of the independent variables were estimated to check whether multicollinearity exists in the models. All the VIFs are below 2, suggesting that multicollinearity is not a significant concern.

All statistical tests were two-tailed with a significance level of *p* < 0.05, except for the interaction analyses where a slightly looser *p*-value cut-off (i.e., *p* < 0.1) was adopted as analyses with interaction terms tend to have a lower statistical power [43].

## 3. Results

### 3.1. Background Characteristics

Table 1 presents descriptive statistics and t/chi-square tests comparing old Chinese men and women. There were moderately more men (52.45%) than women (47.55%). About one-third of participants were in each of the three age groups: 65–69 (33.99%), 70–74 (34.98%), and 75 or older (31.03%). There was no significant difference in the distribution of age groups between men and women. About three-fourths of participants were married/cohabited (72.52%), and more than one-fifth were widowed (22.90%). Women were significantly more likely than men to experience widowhood (39.62% vs. 7.75%).

### 3.2. Sex Differences in Subjective Social Status and Area-Level Deprivation

Men reported significantly higher SES in terms of education and maximum lifetime income. While four out of ten (40.12%) men had secondary or tertiary education, less than 20% (18.23%) of women had such an education level. More than a quarter (26.22%) of men had a maximum lifetime income over HKD 15000, more than seven times that income level among women (3.62%). Additionally, more women reported a high level of area deprivation than men (55.23% vs. 47.87%, *p* < 0.001). While women had lower education and lower income and tended to live in areas with higher deprivation, they reported higher perceived rank on both the society ladder and community ladder than men. Such results indicated a discrepancy between objective measures of SES and subjective social status.

### 3.3. Sex Differences in Health Outcomes

As shown in Table 1, men had better physical performance measured by grip strength and reported higher health-related quality of life in both physical and mental domains. There was no significant sex difference in depression. Our results showed a higher prevalence of depression among women (22.3%) than men (21.5%). However, the sex difference in depression was not statistically significant. Several studies using self-report data have documented an inverted U-shaped function for the sex difference in the initial diagnosis of depression throughout the lifespan, with the sex difference in depression emerging between the ages of 11 and 15, increasing into adulthood, and becoming smaller and perhaps even disappeared altogether in older adults [44,45]. Given our study included subjects aged 65 and above, the sex difference in depression might not be discernible.

### 3.4. Associations between Subjective Social Status, Area Deprivation, and Health

Table 2 shows the main effect of two social ladders and area deprivation on each of the four dependent variables among the full sample after adjusting for sociodemographic variables and objective measures of SES (i.e., education, income). Higher perceived rank on the society ladder and community ladder were associated with lower depression (society ladder: ORa = 0.83, 95% CI = [0.79, 0.87], *p* < 0.001; community ladder: ORa = 0.82, 95% CI = [0.79, 0.86], *p* < 0.001) and higher health-related quality of life in the mental domain (society ladder: b = 0.15, 95% CI = [0.02, 0.29], *p* < 0.05; community ladder: b = 0.33, 95% CI = [0.22, 0.45], *p* < 0.001) and physical domain (society ladder: b = 0.36, 95% CI = [0.21, 0.51], *p* < 0.001; community ladder: b = 0.43, 95% CI = [0.30, 0.56], *p* < 0.001). Only perceived rank on the community ladder was positively associated with physical health among the older people (b = 0.16, 95% CI = [0.07, 0.25], *p* < 0.001). Unexpectedly, the area-level social deprivation index was not associated with any of the four health outcomes.

In addition, some measures of objective socioeconomic status were significantly associated with health outcomes among older people. For example, participants with university education or above were less likely to experience depression (ORa = 0.62, 95% CI = [0.42, 0.91], *p* < 0.05) and had higher health-related quality of life in the mental domain (b = 1.09, 95% CI = [0.10, 2.08], *p* < 0.05) than those with no formal education. Participants with a maximum lifetime income of HKD 15,000 to HKD 29,999 (b = 1.43, 95% CI = [0.81, 2.06], *p* < 0.001) and HKD 30,000 or over (b = 0.98, 95% CI = [0.16, 1.79], *p* < 0.05) tended to have higher grip strength than those with HKD 15000 or below.

### 3.5. Sex Differences in the Associations between Subjective Social Status, Area Deprivation, and Health

**Figure 1:**
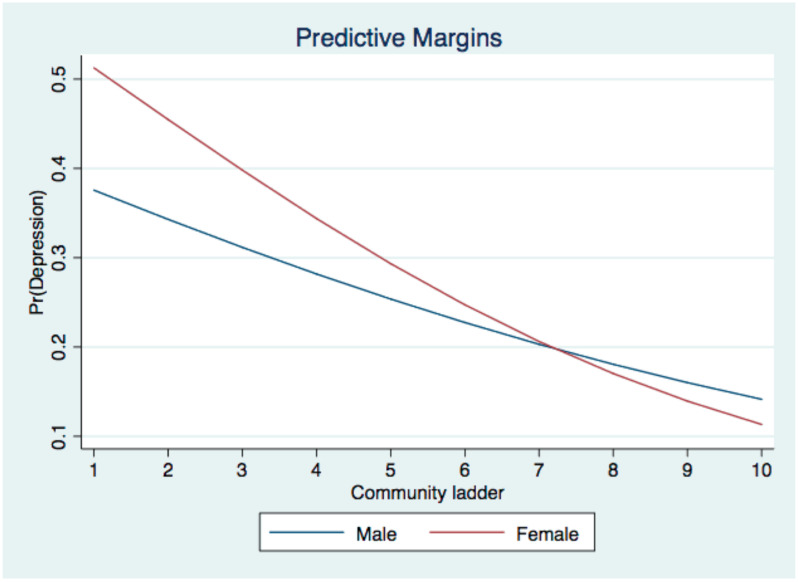
Slopes for men and women.

Table 3 shows the interactive effect between sex and two social ladders and area deprivation index on health outcomes among Chinese older people. There was only one significant interaction, that is, between sex and community ladder on depression (Ora = 0.91; 95% CI = [0.84, 0.99], *p* < 0.05), which suggested that the negative association between community ladder and depression was stronger among women. The significant interactive effect was graphically demonstrated in Figure 1.

### 3.6. Interaction between Subjective Social Status and Area Deprivation 

**Figure 2:**
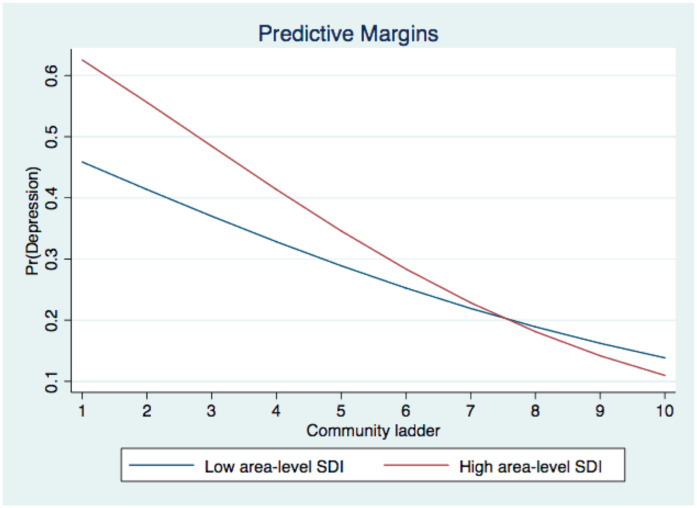
Interactive effect between community ladder and area-level SDI on depression among women.

Table 4 presents the results of four health outcomes on the interactive effects between subjective social status and area-level social deprivation. Models 1a to 4a show the results among the full sample, while Models 1b to 4b and Models 1c to 4c show the results among men and women, respectively. Among the full sample, no significant interaction existed between subjective social status and area-level social deprivation. However, subgroup analysis revealed that the interaction between community ladder and area-level SDI was significant among women (Model 1c: ORa = 0.90, 95% CI = [0.80, 1.01], *p* = 0.07), but not among men. The results suggested that for women, the negative association between relative status in the community and depression was strengthened among those living in more deprived districts. The significant interactive effect is graphically demonstrated in Figure 2.

**Figure 3:**
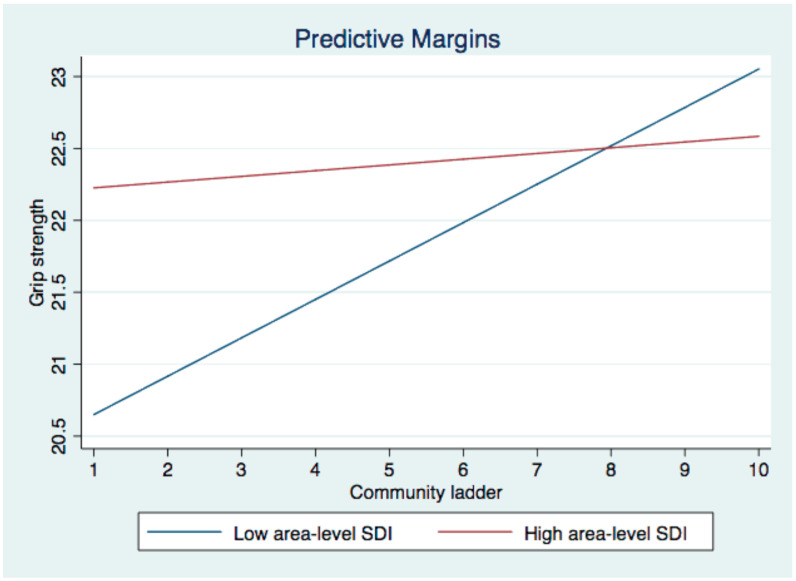
Interactive effect between SES ladder-community and SDI on grip strength among women.

Additionally, the interaction between perceived status in the community and area-level social deprivation was significant for grip strength among women (Model 2c: logit = −0.23, 95%CI = [−0.43, −0.02], *p* < 0.05). The simple slope analysis revealed that perceived status in the community was only positively associated with grip strength among women living in districts with lower social deprivation (Figure 3). 

## 4. Discussion

This study was among the first to consider the unique impact of subjective social status and area-level social deprivation on physical and mental health among old Chinese people and the sex differences in these associations. The aim of this paper was three-fold. First, it assessed the main effect of subjective social status and area-level deprivation on physical and mental health among older Chinese in Hong Kong. Our findings reaffirm that subjective social status is an independent indicator of health after adjusting for objective SES measures (e.g., education and income). Participants who perceived that they were in lower rungs of subjective social status tended to experience more symptoms of depression, have a lower quality of life, and have lower grip strength. Such findings contribute to research on health inequality during later life-course stages when the evidence on the size and patterns of health inequalities in old age is ambiguous [46]. The subjective social status of older people does not only reflect their current living conditions but also their perceptions of their past experiences and future prospects, which is essential to their health.

Moreover, our results revealed that the perceived rank on the community ladder seems to be a more important determinant of health among older people than the society ladder. While both ladders are associated with mental health and health-related quality of life among older Chinese, only the perceived rank on the community ladder was associated with their physical health. Prior evidence regarding which ladder matters more for health outcomes has been inconsistent. Some studies have shown a smaller effect of the community ladder on health than the society ladder (e.g., [47,48]), whereas other studies revealed that the community ladder associations with health are as large (e.g., [49]) or larger than the society ladder (e.g., [50,51]). Given older adults are mostly retired and spend more time in their communities, perceived status in their immediate community may better reflect their relative position and life conditions than status in the larger and less immediate national population. As a result, relative standing in the community may play a more significant role in determining the health status of older people.

In contrast, we found that area deprivation level was not a significant correlate of health among older adults in Hong Kong. Such a finding is consistent with past literature showing that the relationship between individual-level SES and health seems to be more pronounced than small area deprivation [21]. Hong Kong does not have the equivalent of area deprivation as in other countries, likely because public and private buildings are quite often next to each other. It may also be due to a relatively narrow range and a low average level of area deprivation in our sample. Additionally, some scholars argued that how individuals feel about the physical and social environment where they live may be more strongly associated with their mental health than objective measures of area deprivation. Researchers may conduct further studies to explore how perceptions of deprivation affect residents’ health and well-being.

Second, we assessed the interactive effect between subjective social status and area deprivation on the health of older Chinese. While area-level social deprivation had no significant effect on health, it appeared to moderate the impact of subjective social status on health. Our results showed significant interactions between perceived rank on the community ladder and area deprivation on depression and grip strength among women but not among men. It may be because women are more likely to use health and social services in the district than men. The level of deprivation of the districts thus may moderate the relationship between subjective social status and health among women. Specifically, women living in more deprived districts were more likely to report depression if they perceived a lower status in the community. It seems to be consistent with the double jeopardy argument that low-social status people living in disadvantaged areas may be exposed to more stress and have fewer coping resources. In contrast, more better-off districts seemed to buffer the effect of low perceived status in the community on depression among women. However, perception of status in the community was associated with grip strength more strongly among women living in districts with lower social deprivation. Such findings suggested that the interaction between individuals’ perceived community status and area-level deprivation may influence their physical and mental health through somewhat different pathways. 

Third, this study examined the sex differences in the impact of subjective social status and area deprivation on health. Our results suggested that subjective social status was more strongly associated with health in women than in men, although the mechanism behind this remains unclear. Specifically, older women who reported a lower status in their community tended to experience more depressive symptoms than men perceiving the same level of status in the community. However, the interaction between perceived status in society and sex was not significant. In other words, the association between community-referenced subjective status and health differed by sex, whereas the impact of national-referenced status was similar between the two sexes. The stronger correlation between perceived status in the community and mental health occurs among women as opposed to men concurs with previous research showing that deprivation and relative position predict health through psychosocial and interpersonal pathways [11]. Given that women tend to be more sensitive to affiliation concerns than men, social standing in one’s community that involves interpersonal relationships and comparative stress may have a greater impact on women’s psychological well-being.

In addition, our results indicated that men and women may evaluate their place in the social hierarchy differently. We found that women, on average, reported higher subjective social status in both the community and society ladders, despite their lower mean education and income than men. Prior studies suggested that predictors for subjective social status seem to be different between men and women. Men tend to place more weight on their income when ranking their relative status, while women place more weight on their household’s financial standing [52]. The unexpected finding may also be due to women’s significant role in the family in Chinese society, regardless of their educational or income level [53]. Furthermore, it is possible that men have a greater ambition to reach higher goals in terms of income and career, leading to fewer men seeing themselves as having reached such a high position [53]. It may be worthwhile to explore potential causes of the sex differences in conceptualizing and assessing social status in the social hierarchy and the varying strengths of subjective social status in relation to health between men and women in future studies.

The study has several limitations. First, causal relationships between variables cannot be ascertained since we used cross-sectional data. Additional longitudinal studies are essential to determine the causal relationship between subjective social status and health. Second, self-selection bias may exist because those who agreed to take part in the study might be healthier. Third, our measure of area deprivation was based on the district level, which involves large and potentially diverse areas. Such a choice was restricted by data availability, as most indicators for calculating area deprivation were only available at the district level. Future studies may compute area deprivation using smaller geographic areas, e.g., neighborhoods, which reflect better homogeneity and reduce the risk of ecological fallacy. Moreover, despite the significant sex difference in SDI and the self-rating in the society ladder, the means and standard deviations were very similar in the two groups. Some statistically significant differences may not be clinically/practically significant as it also depends on sample size. The absolute difference and the *p*-value should be considered together for a better interpretation. Lastly, although we have adjusted for a set of sociodemographic and socioeconomic factors, such as age, sex, marital status, education, and income, there could be other possibly confounding variables, such as genetic factors. However, these variables were not assessed in the survey. Future studies should include more potential confounders. 

The sex differences in health outcomes among older adults confirm the importance of further work in understanding sex health inequalities. Questions such as whether the basis is entirely biological, cultural, a result of accumulated life course conditions, and the status of women in society, other than education and income, would need to be addressed. A clearer understanding of underlying contributing factors would be important in formulating preventive and community supportive measures. For example, in Hong Kong (and perhaps in other Asian cultures), women have jobs outside of the home and careers to pursue. They are also regarded as being responsible for the upbringing of children, taking on carer roles for older relatives, and running the household.

## 5. Conclusions

In conclusion, our study shows that subjective social status contributes more to health inequalities (both psychological and functional health) than area deprivation indicators among older people. While low subjective social status was strongly associated with worse physical and mental health in older adults after adjusting for objective measures of SES, area-level social deprivation was not significantly associated with the health of older people in Hong Kong. Moreover, perceived rank on the community ladder was more closely related to health among older people than was the society ladder, especially for women. Additionally, we found significant sex differences in health outcomes and in the effect of subjective social status on health. The relative status in the community was more influential on the mental health of women than men. Although area-level social deprivation was not significantly associated with the health of older people, it may moderate the effect of subjective social status on health among women. Women with a lower perceived status in the community were more likely to experience depressive symptoms but better grip strength when living in more deprived neighborhoods. These findings suggested that self-report of subjective social status may provide information about which older adults are at high risk for physical and mental problems. 

It is necessary to raise the perception of social status among older people in order to improve their health and reduce the social gradient in the health of older people, especially among women. Studies have shown that older people’s involvement in decision-making leads to improved self-esteem and a stronger sense of accomplishment [54,55], which may increase their subjective social status. Promoting the participation of older people, particularly women, in policy formulation at all levels of society would be helpful in increasing their subjective social status. Moreover, taking on multiple social roles such as grandparenthood, employment, and volunteering can enhance a person’s subjective social status [56]. However, given there are cultural differences in role experiences, interventions to raise older people’s subjective social status should take into account cultural factors. Support services and networks for older people, as well as carer support, are also vital, particularly in deprived neighborhoods. In addition, raising health literacy about the age-related decline and promoting group activities could benefit older adults’ health and well-being in general.

## Figures and Tables

**Table 1:** Sample characteristics.

	Full Sample (*n* = 3716)		Women (*n* = 1767)		Men (*n* = 1949)		Comparison between Men and Women
	*n*/Mean	%/SD	*n*/Mean	%/SD	*n*/Mean	%/SD	t/Chi-Square
Sex, *n* (%)							
Women	1767	(47.55)	/		/		
Men	1949	(52.45)	/		/		
Age, *n* (%)							
65–69	1263	(33.99)	609	(34.47)	654	(33.56)	0.64
70–74	1300	(34.98)	607	(34.35)	693	(35.56)	
75+	1153	(31.03)	551	(31.18)	602	(30.89)	
Marital status, *n* (%)							
Single	85	(2.29)	44	(2.49)	41	(2.10)	555.74 ***
Married/cohabit	2695	(72.52)	974	(55.12)	1721	(88.30)	
Divorced/separated	85	(2.29)	49	(2.77)	36	(1.85)	
Widowed	851	(22.90)	700	(39.62)	151	(7.75)	
Education, *n* (%)							
No education	713	(19.19)	618	(34.97)	95	(4.87)	361.81 ***
Some primary school	1241	(33.40)	581	(32.88)	660	(33.86)	
Primary school	658	(17.71)	246	(13.92)	412	(21.14)	
Secondary/matriculation	724	(19.48)	209	(11.83)	515	(26.42)	
University or above	380	(10.23)	113	(6.40)	267	(13.70)	
Max. lifetime income, *n* (%)							
Below HKD 15,000	3141	(84.53)	1703	(96.38)	1438	(73.78)	413.34 ***
HKD 15,000 to HKD 29,999	362	(9.74)	40	(2.26)	322	(16.52)	
HKD 30,000 or over	213	(5.73)	24	(1.36)	189	(9.70)	
Area-level social deprivation index (SDI), *n* (%)	1.97	0.07	1.98	0.08	1.97	0.07	24.68 ***
Low area-level SDI	1807	(48.63)	791	(44.77)	1016	(52.13)	
High area-level SDI	1909	(51.47)	976	(55.23)	933	(47.87)	
Society ladder, Mean (SD)	4.56	1.89	4.67	1.91	4.46	1.87	−3.31 ***
Community ladder, Mean (SD)	6.82	2.21	7.34	2.08	6.36	2.22	−13.94 ***
Depression, *n* (%)							
Yes	813	(21.88)	394	(22.30)	419	(21.50)	0.35
No	2903	(78.12)	1373	(77.70)	1530	(78.50)	
Grip strength, Mean (SD)	28.47	(8.15)	22.40	(4.46)	33.98	(6.71)	61.29 ***
Quality of life (physical domain), Mean (SD)	48.80	(8.33)	46.81	(8.70)	50.59	(7.54)	14.22 ***
Quality of life (mental domain), Mean (SD)	55.47	(7.21)	55.09	(7.65)	55.81	(6.76)	3.03 **

** *p* < 0.01, and *** *p* < 0.001.

**Table 2:** Associations between subjective social status, area deprivation, and health outcomes.

	Depression		Grip Strength		Quality of Life (Mental Domain)		Quality of Life (Physical Domain)	
	ORa	[95% CI]	b	[95% CI]	b	[95% CI]	b	[95% CI]
Society ladder	0.83 ***	[0.79, 0.87]	0.07	[−0.03, 0.17]	0.15 *	[0.02, 0.29]	0.36 ***	[0.21, 0.51]
Community ladder	0.82 ***	[0.79, 0.86]	0.16 ***	[0.07, 0.25]	0.33 ***	[0.22, 0.45]	0.43 ***	[0.30, 0.56]
Area-level SDI								
Low area-level SDI	Ref		Ref		Ref		Ref	
High area-level SDI	0.99	[0.84, 1.17]	0.03	[−0.32, 0.38]	0.19	[−0.28, 0.65]	−0.05	[−0.57, 0.47]
Education								
No education	Ref		Ref		Ref		Ref	
Some primary school	0.90	[0.72, 1.14]	−0.42	[−0.95, 0.10]	1.04 **	[0.35, 1.73]	0.51	[−0.27, 1.28]
Primary school	0.81	[0.61, 1.07]	0.17	[−0.44, 0.78]	0.39	[−0.41, 1.19]	0.82	[−0.08, 1.72]
Secondary/matriculation	0.80	[0.60, 1.07]	−0.07	[−0.68, 0.55]	1.30 **	[0.48, 2.11]	0.82	[−0.10, 1.73]
University or above	0.62 *	[0.42, 0.91]	0.53	[−0.22, 1.28]	1.09 *	[0.10, 2.08]	1.05	[−0.06, 2.16]
Max. lifetime income								
Below HKD 15,000	Ref		Ref		Ref		Ref	
HKD 15,000 to HKD 29,999	0.73	[0.53, 1.00]	1.43 ***	[0.81, 2.06]	−0.03	[−0.85, 0.80]	0.57	[−0.35, 1.49]
HKD 30,000 or over	0.68	[0.42, 1.10]	0.98 *	[0.16, 1.79]	0.85	[−0.23, 1.92]	0.58	[−0.62, 1.79]
Sex								
Men	Ref		Ref		Ref		Ref	
Women	1.09	[0.89, 1.33]	−11.26 ***	[−11.69, −10.84]	−0.59 *	[−1.15, −0.03]	−4.10 ***	[−4.73, −3.47]
Age								
65–69	Ref		Ref		Ref		Ref	
70–74	0.91	[0.74, 1.11]	−1.68 ***	[−2.10, −1.25]	0.24	[−0.32, 0.80]	−0.08	[−0.70, 0.55]
75+	1.05	[0.85, 1.30]	−4.09 ***	[−4.54, −3.63]	0.70 *	[0.10, 1.30]	−1.16 ***	[−1.84, −0.49]
Marital status								
Single	Ref		Ref		Ref		Ref	
Married/cohabit	0.57 *	[0.35, 0.93]	1.93 **	[0.75, 3.10]	0.97	[−0.58, 2.52]	−0.73	[−2.46, 1.01]
Divorced/separated	1.12	[0.57, 2.22]	−0.22	[−1.86, 1.41]	0.33	[−1.82, 2.48]	−0.39	[−2.80, 2.02]
Widowed	0.68	[0.41, 1.14]	1.47 *	[0.26, 2.68]	0.60	[−1.00, 2.20]	−0.10	[−1.89, 1.70]

ORa = adjusted odds ratio; * *p* < 0.05, ** *p* < 0.01, and *** *p* < 0.001.

**Table 3:** Associations between perceived social status, area deprivation, and sex differences in health outcomes.

	Depression		Grip Strength		Quality of Life (Mental Domain)		Quality of Life (Physical Domain)	
	ORa	[95% CI]	b	[95% CI]	b	[95% CI]	b	[95% CI]
Society ladder	0.80 ***	[0.74, 0.86]	0.08	[−0.11, 0.27]	0.03	[−0.12, 0.17]	0.46 ***	[0.25, 0.67]
Community ladder	0.86 ***	[0.81, 0.91]	0.41 ***	[0.26, 0.57]	0.16 **	[0.04, 0.28]	0.34 ***	[0.16, 0.51]
High area-level SDI	0.92	[0.73, 1.16]	0.30	[−0.33, 0.94]	−0.14	[−0.62, 0.34]	0.26	[−0.46, 0.97]
Women	1.28	[0.71, 2.30]	0.02	[−1.73, 1.78]	−11.84 ***	[−13.17, −10.51]	−4.26 ***	[−6.23, −2.30]
Society ladder × women	1.08	[0.98, 1.19]	0.14	[−0.13, 0.40]	0.08	[−0.12, 0.28]	−0.20	[−0.49, 0.09]
Community ladder × women	0.91 *	[0.84, 0.99]	−0.16	[−0.39, 0.07]	0.00	[−0.17, 0.18]	0.21	[−0.05, 0.46]
High area-level SDI × women	1.18	[0.85, 1.64]	−0.24	[−1.17, 0.69]	0.35	[−0.35, 1.06]	−0.66	[−1.69, 0.38]

ORa = adjusted odds ratio; all models adjusted for age, education, marital status, and max. lifetime income. * *p* < 0.05, ** *p* < 0.01, and *** *p* < 0.001.

**Table 4:** Interactive effects between subjective social status and area deprivation in men and women.

	Depression						Grip Strength					
	Model 1a		Model 1b		Model 1c		Model 2a		Model 2b		Model 2c	
	Full Sample		Men		Women		Full Sample		Men		Women	
	ORa	[95% CI]	ORa	[95% CI]	ORa	[95% CI]	b	[95% CI]	b	[95% CI]	b	[95% CI]
Society ladder	0.82 ***	[0.76, 0.88]	0.78 ***	[0.71, 0.86]	0.86 **	[0.78, 0.96]	0.06	[−0.08, 0.21]	0.09	[−0.14, 0.32]	0.04	[−0.13, 0.21]
Community ladder	0.85 ***	[0.80, 0.90]	0.87 ***	[0.81, 0.94]	0.83 ***	[0.76, 0.90]	0.22 ***	[0.09, 0.34]	0.18	[−0.01, 0.37]	0.27 ***	[0.12, 0.41]
High area-level SDI	1.32	[0.76, 2.27]	1.01	[0.49, 2.09]	2.24	[0.94, 5.36]	0.73	[−0.50, 1.97]	0.91	[−0.94, 2.76]	1.09	[−0.47, 2.66]
Society ladder × high area-level SDI	1.02	[0.93, 1.13]	1.03	[0.89, 1.18]	1.00	[0.87, 1.15]	0.01	[−0.19, 0.21]	−0.18	[−0.50, 0.14]	0.15	[−0.07, 0.37]
Community ladder × high area-level SDI	0.94	[0.87, 1.02]	0.97	[0.87, 1.08]	0.90 ^#^	[0.80, 1.01]	−0.11	[−0.28, 0.06]	−0.04	[−0.31, 0.23]	−0.23 *	[−0.43, −0.02]
	**Quality of Life (Mental Domain)**						**Quality of Life (Physical Domain)**					
	**Model 3a**		**Model 3b**		**Model 3c**		**Model 4a**		**Model 4b**		**Model 4c**	
	**Full Sample**		**Men**		**Women**		**Full Sample**		**Men**		**Women**	
	**b**	**[95% CI]**	**b**	**[95% CI]**	**b**	**[95% CI]**	**b**	**[95% CI]**	**b**	**[95% CI]**	**b**	**[95% CI]**
Society ladder	0.10	[−0.09, 0.29]	0.08	[−0.17, 0.33]	0.12	[−0.18, 0.43]	0.34 **	[0.12, 0.55]	0.56 ***	[0.29, 0.84]	0.07	[−0.27, 0.42]
Community ladder	0.42 ***	[0.26, 0.58]	0.48 ***	[0.28, 0.68]	0.35 **	[0.09, 0.60]	0.45 ***	[0.27, 0.63]	0.27 *	[0.04, 0.49]	0.62 ***	[0.33, 0.91]
High area-level SDI	0.91	[−0.72, 2.54]	1.31	[−0.67, 3.30]	0.40	[−2.41, 3.21]	0.02	[−1.80, 1.84]	0.13	[−2.07, 2.33]	−0.46	[−3.63, 2.70]
Society ladder × high area-level SDI	0.10	[−0.16, 0.36]	−0.01	[−0.35, 0.34]	0.19	[−0.21, 0.59]	0.04	[−0.26, 0.33]	−0.19	[−0.58, 0.19]	0.30	[−0.14, 0.75]
Community ladder × High area-level SDI	−0.17	[-0.39,0.05]	−0.15	[−0.44,0.14]	−0.17	[−0.53,0.19]	−0.03	[−0.28,0.22]	0.15	[−0.17,0.48]	−0.18	[−0.59,0.23]

ORa = adjusted odds ratio; all models adjusted for age, education, marital status, and max. Lifetime income. ^#^ *p* < 0.1, * *p* < 0.05, ** *p* < 0.01, and *** *p* < 0.001.

## Data Availability

The data are available upon reasonable request from the corresponding author.

## References

[1] Adler N.E., Rehkopf D.H. (2008). US disparities in health: Descriptions, causes, and mechanisms. Annu. Rev. Public Health.

[2] Chetty R., Stepner M., Abraham S., Lin S., Scuderi B., Turner N., Bergeron A., Cutler D. (2016). The association between income and life expectancy in the United States, 2001-2014. JAMA.

[3] Chen F., Yang Y., Liu G. (2010). Social change and socioeconomic disparities in health over the life course in China: A cohort analysis. Am. Sociol. Rev..

[4] Lei X., Sun X., Strauss J., Zhang P., Zhao Y. (2014). Depressive symptoms and SES among the mid-aged and elderly in China: Evidence from the China Health and Retirement Longitudinal Study national baseline. Soc. Sci. Med..

[5] Euteneuer F. (2014). Subjective social status and health. Curr. Opin. Psychiatry.

[6] Zell E., Strickhouser J.E., Krizan Z. (2018). Subjective social status and health: A meta-analysis of community and society ladders. Health Psychol..

[7] Singh-Manoux A., Marmot M.G., Adler N.E. (2005). Does subjective social status predict health and change in health status better than objective status?. Psychosom. Med..

[8] Demakakos P., Nazroo J., Breeze E., Marmot M. (2008). Socioeconomic status and health: The role of subjective social status. Soc. Sci. Med..

[9] Kwong E., Kwok T.T., Sumerlin T.S., Goggins W.B., Leung J., Kim J.H. (2020). Does subjective social status predict depressive symptoms in Chinese elderly? A longitudinal study from Hong Kong. J. Epidemiol. Community Health.

[10] Chen B., Covinsky K.E., Cenzer I.S., Adler N., Williams B.A. (2012). Subjective social status and functional decline in older adults. J. Gen. Intern. Med..

[11] Jin L., Tam T. (2015). Investigating the effects of temporal and interpersonal relative deprivation on health in China. Soc. Sci. Med..

[12] Adler N.E., Epel E.S., Castellazzo G., Ickovics J.R. (2000). Relationship of subjective and objective social status with psychological and physiological functioning: Preliminary data in healthy, White women. Health Psychol..

[13] Woo J., Leung J., Chan R., Chau P. (2013). Influence of income and self-rated socio-economic position on lifestyle, and physical and psychological function in older Chinese adults aged 65 years and over. Public Health.

[14] Adler N., Singh-Manoux A., Schwartz J., Stewart J., Matthews K., Marmot M.G. (2008). Social status and health: A comparison of British civil servants in Whitehall-II with European-and African-Americans in CARDIA. Soc. Sci. Med..

[15] Mutyambizi C., Booysen F., Stornes P., Eikemo T.A. (2019). Subjective social status and inequalities in depressive symptoms: A gender-specific decomposition analysis for South Africa. Int. J. Equity Health.

[16] Singh-Manoux A., Adler N.E., Marmot M.G. (2003). Subjective social status: Its determinants and its association with measures of ill-health in the Whitehall II study. Soc. Sci. Med..

[17] Lundberg J., Kristenson M. (2008). Is subjective status influenced by psychosocial factors?. Soc. Indic. Res..

[18] Takahashi Y., Fujiwara T., Nakayama T., Kawachi I. (2018). Subjective social status and trajectories of self-rated health status: A comparative analysis of Japan and the United States. J. Public Health.

[19] Jansen L., Eberle A., Emrich K., Gondos A., Holleczek B., Kajüter H., Maier W., Nennecke A., Pritzkuleit R., Brenner H. (2014). Socioeconomic deprivation and cancer survival in Germany: An ecological analysis in 200 districts in Germany. Int. J. Cancer.

[20] Maier W., Scheidt-Nave C., Holle R., Kroll L.E., Lampert T., Du Y., Heidemann C., Mielck A. (2014). Area level deprivation is an independent determinant of prevalent type 2 diabetes and obesity at the national level in Germany. Results from the National Telephone Health Interview Surveys ‘German Health Update’ GEDA 2009 and 2010. PLoS ONE.

[21] Siegel M., Mielck A., Maier W. (2015). Individual income, area deprivation, and health: Do income-related health inequalities vary by small area deprivation?. Health Econ..

[22] Ostir G.V., Eschbach K., Markides K.S., Goodwin J.S. (2003). Neighbourhood composition and depressive symptoms among older Mexican Americans. J. Epidemiol. Community Health.

[23] Kubzansky L.D., Subramanian S., Kawachi I., Fay M.E., Soobader M.-J., Berkman L.F. (2005). Neighborhood contextual influences on depressive symptoms in the elderly. Am. J. Epidemiol..

[24] Yen I.H., Michael Y.L., Perdue L. (2009). Neighborhood environment in studies of health of older adults: A systematic review. Am. J. Prev. Med..

[25] Gale C.R., Dennison E.M., Cooper C., Sayer A.A. (2011). Neighbourhood environment and positive mental health in older people: The Hertfordshire Cohort Study. Health Place.

[26] Stafford M., Marmot M. (2003). Neighbourhood deprivation and health: Does it affect us all equally?. Int. J. Epidemiol..

[27] Boylan J.M., Robert S.A. (2017). Neighborhood SES is particularly important to the cardiovascular health of low SES individuals. Soc. Sci. Med..

[28] Wang J.-J., Snyder M., Kaas M. (2001). Stress, loneliness, and depression in Taiwanese rural community-dwelling elders. Int. J. Nurs. Stud..

[29] Kawachi I., Kennedy B.P. (1999). Income inequality and health: Pathways and mechanisms. Health Serv. Res..

[30] Woo J., Tang N., Suen E., Leung J., Leung P. (2008). Telomeres and frailty. Mech. Ageing Dev..

[31] Woo J., Leung J., Lau E. (2009). Prevalence and correlates of musculoskeletal pain in Chinese elderly and the impact on 4-year physical function and quality of life. Public Health.

[32] Woo J., Leung J. (2018). Sarcopenic obesity revisited: Insights from the Mr and Ms Os cohort. J. Am. Med. Dir. Assoc..

[33] Yu R., Wong M., Leung J., Lee J., Auyeung T.W., Woo J. (2014). Incidence, reversibility, risk factors and the protective effect of high body mass index against sarcopenia in community-dwelling older C hinese adults. Geriatr. Gerontol. Int..

[34] Helgeson V.S., Suls J., Wallston K.A. (2003). Gender-related traits and health. Social Psychological Foundations of Health and Illness.

[35] Smith T.W., Gallo L.C., Goble L., Ngu L.Q., Stark K.A. (1998). Agency, communion, and cardiovascular reactivity during marital interaction. Health Psychol..

[36] Wong S., Kwok T., Woo J., Lynn H., Griffith J., Leung J., Tang Y., Leung P. (2005). Bone mineral density and the risk of peripheral arterial disease in men and women: Results from Mr. and Ms Os, Hong Kong. Osteoporos. Int..

[37] The MacArthur Scale of Subjective Social Status. http://www.macses.ucsf.edu/research/psychosocial/subjective.php.

[38] Wang K., Law C.-K., Zhao J., Hui A.Y.-K., Yip B.H.-K., Yeoh E.K., Chung R.Y.-N. (2021). Measuring health-related social deprivation in small areas: Development of an index and examination of its association with cancer mortality. Int. J. Equity Health.

[39] Hong Kong Census and Statistics Department 2016 Population by-Census. https://www.bycensus2016.gov.hk/en/.

[40] de Craen A.J., Heeren T., Gussekloo J. (2003). Accuracy of the 15-item geriatric depression scale (GDS-15) in a community sample of the oldest old. Int. J. Geriatr. Psychiatry.

[41] Weintraub D., Oehlberg K.A., Katz I.R., Stern M.B. (2006). Test characteristics of the 15-item geriatric depression scale and Hamilton depression rating scale in Parkinson disease. Am. J. Geriatr. Psychiatry.

[42] Lam C.L., Tse E.Y., Gandek B. (2005). Is the standard SF-12 health survey valid and equivalent for a Chinese population?. Qual. Life Res..

[43] Twisk J.W. (2019). Applied Mixed Model Analysis: A Practical Guide.

[44] Gutiérrez-Lobos K., Scherer M., Anderer P., Katschnig H. (2002). The influence of age on the female/male ratio of treated incidence rates in depression. BMC Psychiatry.

[45] Lewinsohn P.M., Duncan E.M., Stanton A.K., Hautzinger M. (1986). Age at first onset for nonbipolar depression. J. Abnorm. Psychol..

[46] Chandola T., Ferrie J., Sacker A., Marmot M. (2007). Social inequalities in self reported health in early old age: Follow-up of prospective cohort study. Bmj.

[47] Correa-Velez I., Gifford S.M., Barnett A.G. (2010). Longing to belong: Social inclusion and wellbeing among youth with refugee backgrounds in the first three years in Melbourne, Australia. Soc. Sci. Med..

[48] Wolff L.S., Subramanian S.V., Acevedo-Garcia D., Weber D., Kawachi I. (2010). Compared to whom? Subjective social status, self-rated health, and referent group sensitivity in a diverse US sample. Soc. Sci. Med..

[49] Subramanyam M.A., Diez-Roux A.V., Hickson D.A., Sarpong D.F., Sims M., Taylor H.A., Williams D.R., Wyatt S.B. (2012). Subjective social status and psychosocial and metabolic risk factors for cardiovascular disease among African Americans in the Jackson Heart Study. Soc. Sci. Med..

[50] Euteneuer F., Mills P.J., Rief W., Ziegler M.G., Dimsdale J.E. (2012). Subjective social status predicts in vivo responsiveness of β-adrenergic receptors. Health Psychol..

[51] Ghaed S.G., Gallo L.C. (2007). Subjective social status, objective socioeconomic status, and cardiovascular risk in women. Health Psychol..

[52] Miyakawa M., Magnusson Hanson L.L., Theorell T., Westerlund H. (2012). Subjective social status: Its determinants and association with health in the Swedish working population (the SLOSH study). Eur. J. Public Health.

[53] Woo J., Lynn H., Leung J., Wong S. (2008). Self-perceived social status and health in older Hong Kong Chinese women compared with men. Women Health.

[54] Wallerstein N. (2006). What is the Evidence on Effectiveness of Empowerment to Improve Health? Health Evidence Network Report.

[55] Zimmerman M. (2000). Empowerment Theory: Psychological, Organizational and Community Levels of Analysis.

[56] Barnett R.C., Hyde J.S. (2001). Women, men, work, and family: An expansionist theory. Am. Psychol..

